# 

**Published:** 1880-10

**Authors:** 


### CONTENTS FOR OCTOBER, 1880.

ORIGINAL COMMUNICATIONS.

ART. I.—Treatment of Orchitis, with Note as to its Varieties, Pathology, Prognosis, Treatment, etc. By G. HALSTED BOYLAND, A. M., M. D. .......... 463

ART. II.—A Peculiarity in the Human Hybrid. By HARVEY L. BYRD, A. M., M. D. .... 466

ART. III.—Treatment of Fracture of the Jaw, with Critical Remarks. By THOMAS BRIAN GUNNING, D. D. S. .......... 468

ART. IV.—Blood-Letting in Disease. By HARVEY L. BYRD, M. D .......... 476

CORRESPONDENCE.

The Chisolm-Reuling Imbroglio .......... 480

EDITORIAL DEPARTMENT.

Occupations Suitable for Females .......... 481
"Who Was It?" .......... 483
Buchanan Redivivus .......... 483
Chian Turpentine .......... 483
Yellow Fever .......... 484
A New Sphygmoscope .......... 484
Mutual Life Associations for Physicians and Dentists .......... 485
American Gynæcological Association .......... 485
Sesqui-Centennial Celebration .......... 487

BIBLIOGRAPHICAL DEPARTMENT.

Index Catalogue of the Library of the Surgeon-General's Office, U. S. Army .......... 487
The Practitioner's Reference Book .......... 488
The Skin in Health and Disease .......... 489
Myopia in its Various Phases .......... 489
Minor Notices .......... 491
The Twentieth Session of the American Dental Association .......... 492
The Johnston Brothers' Excursion .......... 492

HYMENEAL .......... 492

NATALITIAL, NECROLOGICAL .......... 493

EXCERPTA.

Dentists in the Army and Navy .......... 494
Thomas on hearing through the teeth .......... 495
De Lesseps Canal in its relation to Hygiene .......... 496
Action of Benzoate of Soda in Scarlet Fever .......... 498
Stigmata of Maize .......... 500
Iodoform Suppositories for the Urethra and Canal of the Cervix Uteri .......... 501
Dentists in America .......... 502
The Medical Registration Law .......... 503
Therapeutic uses of the Bromides .......... 505
Tracheotomy Superseded .......... 506
Duty of Physicians .......... 507
Selections .......... 509

MORTUARY STATISTICS .......... 510

